# Dietary copper intake and the prevalence of kidney stones among adult in the United States: A propensity score matching study

**DOI:** 10.3389/fpubh.2022.973887

**Published:** 2022-08-30

**Authors:** Weidong Zhu, Chunying Wang, Jianping Wu, Shuqiu Chen, Weipu Mao, Yu Chen, Ming Chen

**Affiliations:** ^1^Department of Urology, Affiliated Zhongda Hospital of Southeast University, Nanjing, China; ^2^Department of Nursing, Zhongda Hospital, Southeast University, Nanjing, China

**Keywords:** kidney stones, dietary copper intake, dose-response curves, propensity score matching, NHANES database

## Abstract

**Background:**

Trace metals are essential trace elements for the human body, but insufficient or excessive levels of metal ions can lead to the development of disease. The purpose of this study was to examine the relationship between dietary intake of copper and the prevalence of kidney stones in U.S. adult population.

**Methods:**

We included data on dietary intake of trace metals from 28,623 adult participants in the National Health and Nutrition Examination Survey (NHANES) database between 2007 and 2018. Multivariate logistic regression and restricted cubic spline (RCS) dose-response curves were used to explore the association between trace metals and kidney stones, and 1:1 propensity score matching (PSM) was performed between the stone formers and non-stone formers to test the validity of the results.

**Results:**

Dose-response curves showed a non-linear negative association between dietary copper intake and kidney stones, and an increase in copper intake reduced the risk of kidney stones. Multivariate logistic regression analysis showed that the odds ratio (95% confidence interval) for kidney stones in each quartile of copper intake compared to the lowest quartile were 0.905 (0.808–1.011, *p* = 0.075), 0.880 (0.785–0.987, *p* = 0.028) and 0.853 (0.756–0.959, *p* = 0.009). In addition, similar conclusions were reached after analysis of PSM in the stone formers and non-stone formers groups.

**Conclusion:**

Dietary copper intake was negatively and non-linearly correlated with kidney stones, which is worthy of further research and application in clinical practice.

## Introduction

Kidney stones are a common urological condition caused by the deposition of mineral crystals in the urine in the kidneys (1, 2). Severe kidney stones can cause symptoms such as severe back pain, nausea, hematuria, and difficulty in urination. Bilateral urinary tract obstruction can lead to uremia, and the atrophy of kidney tissue caused by stones severely affects kidney function and may lead to loss of function of the entire kidney (3, 4). Kidney stones are a complex disease caused by environmental, dietary and genetic factors, with an incidence of over 6–12% and a 5-year recurrence rate of up to 50%, seriously affecting human health (5).

Trace metals are indispensable trace elements for living organisms, but insufficient or excessive levels of metal ions can lead to cell death, which in turn cause abnormalities or diseases in human physiology (6, 7). Some studies have found a relationship between trace metals and the occurrence and development of urinary stones (8–10). However, the role of trace metals in the pathogenesis of kidney stones is unclear. Some studies have reported that dietary zinc intake may be associated with an increased risk of kidney stones; whereas manganese intake may be associated with a reduced risk of kidney stones (11, 12). Copper, a trace metal, is an important cofactor in living organisms, and its redox properties make it both beneficial and toxic to cells (13, 14). As an essential trace element, copper is vital for the growth and development of the brain, bones, and other organs (15). Copper can only be consumed from dietary sources, and nut foods, animal offal, vegetables, and legumes contain high amounts of copper (16). Recent studies have found that intracellular copper accumulation can lead to a novel type of programmed cell death (PCD) called cuproptosis (17). However, the relationship between dietary copper intake and the risk of kidney stones is unclear.

In the current study, we used a large US population survey database, the National Health and Nutrition Examination Survey (NHANES) 2007–2018, to explore the dose-response relationship between copper intake and kidney stone risk, and validated it by propensity score matching (PSM) analysis.

## Materials and methods

### Data sources and preparation

The population for this study was drawn from the NHANES database, a cross-sectional survey designed to assess the health and nutritional status of non-institutionalized civilians in all 50 states and the District of Columbia in the United States (18). Approximately 5,000 individuals were sampled each year through a multilevel stratified probability design, and all participants receive a structured questionnaire at home and a physical examination at a mobile testing center. Data from this representative survey have been published online on a 2-year cycle since 1999. The data files published online are available for public use and can be found on the official website: https://www.cdc.gov/nchs/nhanes/index.htm.

**Figure 1:**
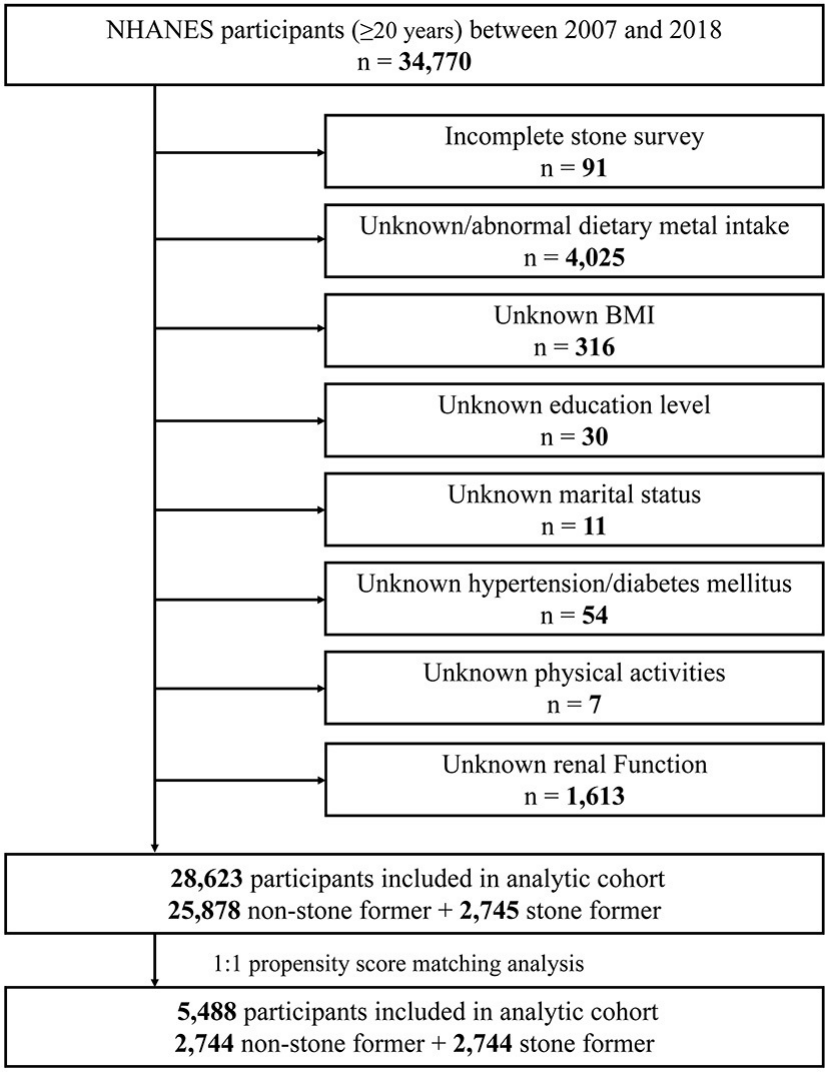
Schematic overview for patient identification.

In this study, we used publicly available data from NHANES for six cycles: 2007–2008, 2009–2010, 2011–2011, 2013–2014, 2015–2016, and 2017–2018. A total of 34,770 adults (≥20 years) participated in the survey during this 12-year period. We developed the following exclusion criteria (Figure 1): (a) incomplete stone survey (*n* = 91); (b) unknown/abnormal dietary trace metal intake (*n* = 4,025); (c) unknown body mass index (BMI) (*n* = 316); (d) unknown education level (*n* = 30); (e) unknown marital status (*n* = 11); (f) unknown hypertension/diabetes mellitus (*n* = 54); (g) unknown physical activities status (*n* = 7); (h) unknown renal function indicators (creatinine, urea nitrogen, and uric acid) (*n* = 1,613). A total of 6,147 individuals were excluded based on the above criteria, and a total of 28,623 study subjects were finally included in this study.

### Study variables

In NHANES, dietary protein, moisture and trace metals (phosphorus, magnesium, iron, zinc, copper, selenium) intake was assessed by the 24 h dietary review method, in which participants were asked by a professional technician about the types and amounts of foods and beverages consumed during 24 h and recorded in the NHANES computer-assisted dietary survey system. The intake of each food component was then estimated based on the University of Texas Food Intake Analysis System and the USDA Survey Nutrient Database.

In addition, we included other variables such as gender, age, race, marital status, family income, education level, BMI, hypertension, diabetes mellitus, smoking status, drinking, physical activities status (vigorous and moderate activities), blood urea nitrogen, creatinine, uric acid, and estimated glomerular filtration rate (eGFR). BMI was calculated as weight (kg)/[height (m^2^)^*^height (m^2^)]. Hypertension and diabetes mellitus were diagnosed by a physician or other health professional. The eGFR was calculated were followed as described previously (3, 19).

### Kidney stones assessment

In the questionnaire, the participant was asked by a trained professional: “Have you ever had kidney stones”, and the participant was considered to have a history of kidney stones if he/she answered “Yes”.

### Statistical analysis

All data and figures in this study were organized and analyzed using R software (version 3.5.3) and SPSS software (version 24.0). Sampling weights for interviews (WTSA2YR) and study design variables (SDMVPSU and SDMVSTRA) were used in the analysis of the data, and the weighting analysis was performed to consider the complex sampling design and to obtain the appropriate weights. Normally distributed continuous data were described by mean ± standard deviation (SD), and categorical data were described by frequency (*n*) and percentage (%). Trace metals were quadratically divided and multivariate logistic regression models were used to assess the association between different dietary trace metal intakes and kidney stones, with results presented as adjusted odds ratios (aORs) and 95% confidence intervals (CIs) intervals. For multivariate logistic regression analysis, we used two models to assess the effect of trace metals on kidney stones: the single-metal model: adjusted for individual trace metals and all other covariates; the multi-metal model: adjusted for all trace metals and all other covariates. Restricted cubic spline (RCS) functions were used to visually describe the dose-response relationship between dietary trace metal intake and kidney stones.

A 1:1 propensity score matching (PSM) analysis was used to balance the differences between the stone forming and non-stone formers, adjusted for confounding variables including: gender, age, race, marital status, family income, education level, BMI, hypertension, diabetes mellitus, smoking status, drinking, vigorous recreational activities and moderate recreational activities. The data after PSM were also reanalyzed to further test the correctness of the results. *P*-values were considered statistically significant when calculated at <0.05.

## Results

A total of 28,623 participants were included in the study between 2007 and 2018, of which 2,745 (9.6%) were stone formers and 25,878 (90.4%) were non-stone formers. The clinicopathological characteristics of all participants are shown in Table 1. The chi-square test showed significant differences between stone formers and non-stone formers on the variables of gender, age, race, marital status, BMI, hypertension, diabetes mellitus, and physical activities variables (all *P* < 0.001). In addition, stone formers had higher levels of blood urea nitrogen, creatinine, and uric acid and lower eGFR compared to non-stone formers. Moreover, the dietary intakes of phosphorus, magnesium, copper and selenium were lower in stone formers compared to non-stone formers (all *P* < 0.05). Spearman's rank correlation coefficient analysis showed a positive correlation between dietary intakes of the six trace metals (Supplementary Figure S1).

**Table 1:** Baseline characteristics of NHANES participants between 2007 and 2018.

**Characteristic**	**Total**	**None-stone formers**	**Stone formers**	***P*** **-value**
	**No. (%)**	**No. (%)**	**No. (%)**	
Total patients	28,623	25,878 (90.4)	2,745 (9.6)	
**Gender**				<0.001
Male	13,924 (48.6)	12,390 (47.9)	1,534 (55.9)	
Female	14,699 (51.4)	13,488 (52.1)	1,211 (44.1)	
**Age**				<0.001
<50 years	14,402 (50.3)	13,451 (52.0)	951 (34.6)	
≥50 years	14,221 (49.7)	12,427 (48.0)	1,794 (65.4)	
**Race**				<0.001
Non-Hispanic white	12,098 (42.3)	10,580 (40.9)	1,518 (55.3)	
Non-Hispanic black	5,891 (20.6)	5,539 (21.4)	352 (12.8)	
Mexican American	4,381 (15.3)	4,027 (15.6)	354 (12.9)	
Other Hispanic	3,005 (10.5)	2,701 (10.4)	304 (11.1)	
**Other**	3,248 (11.3)	3,031 (11.7)	217 (7.9)	
Education level				0.550
Less than high school	6,859 (24.0)	6,178 (23.9)	681 (24.8)	
High school or equivalent	6,541 (22.9)	5,922 (22.9)	619 (22.6)	
College or above	15,223 (53.2)	13,778 (53.2)	1,445 (52.6)	
**Marital status**				<0.001
Married	14,740 (51.5)	13,166 (50.9)	1,574 (57.3)	
Unmarried	13,883 (48.5)	12,712 (49.1)	1,171 (42.7)	
**Family income**				0.198
< $20,000	6,572 (23.0)	5,922 (22.9)	650 (23.7)	
≥$20,000	20,647 (72.1)	18,669 (72.1)	1,978 (72.1)	
Unknown	1,404 (4.9)	1,287 (5.0)	117 (4.3)	
**BMI (kg/m^2^)**				<0.001
<25.0	8,048 (28.1)	7,519 (29.1)	529 (19.3)	
≥25.0	20,575 (71.9)	18,359 (70.9)	2,216 (80.7)	
**Hypertension**				<0.001
Yes	10,336 (36.1)	8,943 (34.6)	1,393 (50.7)	
No	18,287 (63.9)	16,935 (65.4)	1,352 (49.3)	
**Diabetes mellitus**				<0.001
Yes	3,716 (13.0)	3,099 (12.0)	617 (22.5)	
No	24,236 (84.7)	22,202 (85.8)	2,034 (74.1)	
Borderline	671 (2.3)	577 (2.2)	94 (3.4)	
**Smoking status**				<0.001
Never	15,951 (55.7)	14,608 (56.4)	1,343 (48.9)	
Former	6,900 (24.1)	6,045 (23.4)	855 (31.1)	
Current	5,772 (20.2)	5,225 (20.2)	547 (19.9)	
Drinking				0.216
Yes	20,334 (71.0)	18,356 (70.9)	1,978 (72.1)	
No/Unknown	8,299 (29.0)	7,522 (29.1)	767 (27.9)	
**Vigorous recreational activities**				<0.001
Yes	6,306 (22.0)	5,890 (22.8)	416 (15.2)	
No	22,317 (78.0)	19,988 (77.2)	2,329 (84.8)	
**Moderate recreational activities**				<0.001
Yes	11,574 (40.4)	10,590 (40.9)	984 (35.8)	
No	17,049 (59.6)	15,288 (59.1)	1,761 (64.2)	
Blood urea nitrogen (mg/dL)	13.76, 5.99	13.60, 5.81	15.29, 7.27	<0.001
Creatinine (mg/dL)	0.90, 0.45	0.89, 0.41	0.98, 0.72	<0.001
Uric acid (mg/dL)	5.45, 1.45	5.43, 1.44	5.65, 1.52	<0.001
eGFR [mL/(min·1.73 m^2^)]	83.71, 25.80	84.63, 25.65	75.04, 25.61	<0.001
**Daily intake (M, SD)**				
Protein (mg)	80.75, 42.16	81.01, 42.33	78.38, 40.49	0.002
Moisture (g)	2.88, 1.50	2.89, 1.50	2.83, 1.47	0.062
Phosphorus (mg)	1,340.9, 667.7	1,344.4, 670.2	1,307.5, 642.2	0.006
Magnesium (mg)	294.63, 146.58	295.88, 147.25	282.78, 139.54	<0.001
Iron (mg)	14.42, 8.33	14.42, 8.33	14.41, 8.33	0.966
Zinc (mg)	11.04, 6.71	11.06, 6.74	10.87, 6.43	0.157
Copper (mg)	1.23, 0.74	1.24, 0.74	1.20, 0.69	0.007
Selenium (mcg)	112.36, 63.40	112.61, 63.50	110.03, 62.45	0.043

For categorical variables, *P*-values were analyzed by chi-square tests. For continuous variables, the *t*-test for slope was used in generalized linear models.

NHANES, National Health and Nutrition Examination Survey; BMI, body mass index; eGFR, estimated glomerular filtration rate; SD, standard deviation.

**Figure 2:**
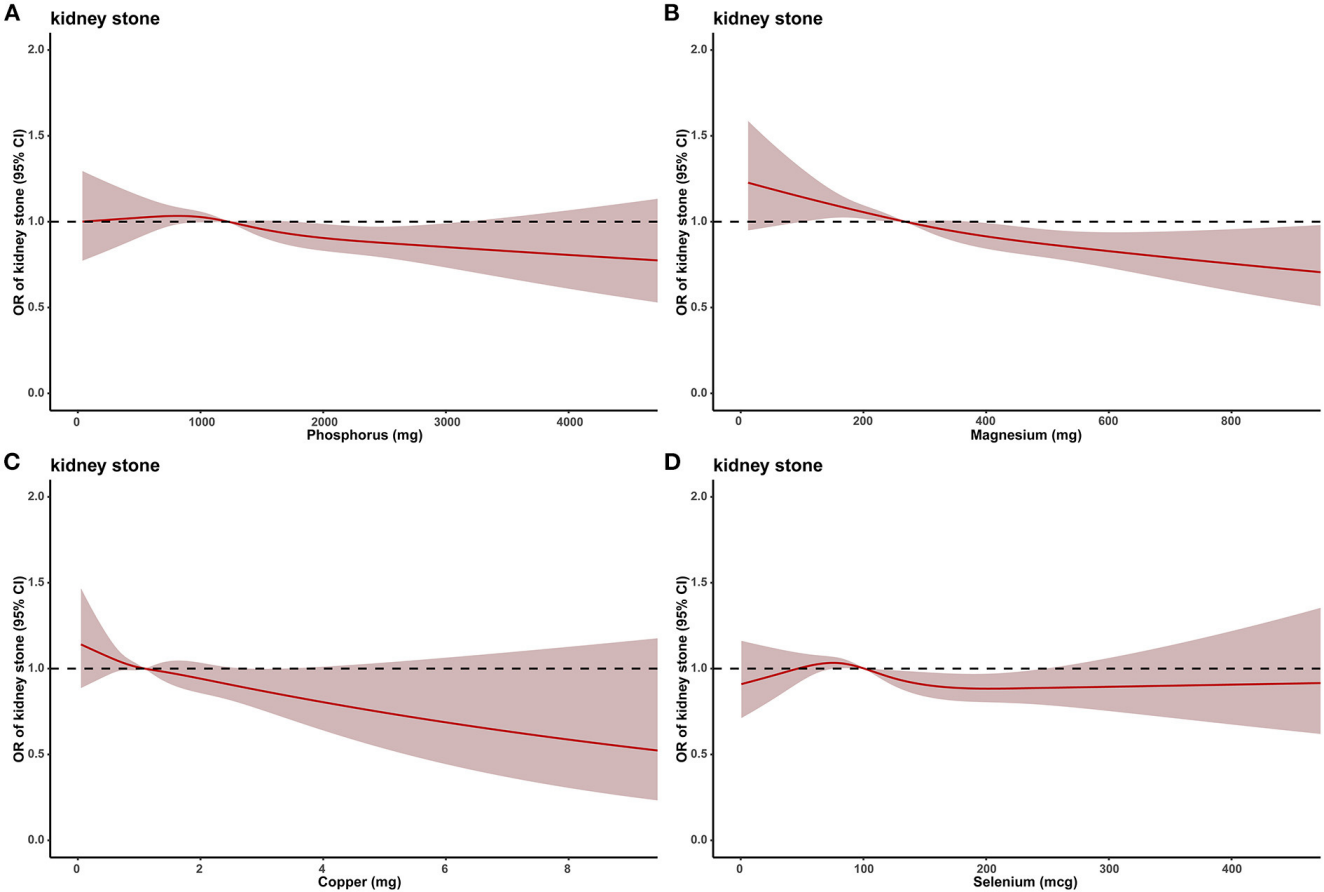
The dose–response analysis between dietary metal intake and presence of kidney stones before propensity score matching. **(A)** Phosphorus. **(B)** Magnesium. **(C)** Copper. **(D)** Selenium.

Dose-response curve analysis of the RCS was used to assess the relationship between dietary phosphorus, magnesium, copper, selenium intakes and kidney stones. The results showed that dietary phosphorus (*p* = 0.038), magnesium (*p* < 0.001), and copper (*p* = 0.002) intakes were negatively and non-linearly associated with kidney stone risk in all populations, and the risk of kidney stones decreased progressively with increasing phosphorus, magnesium, or copper intakes (Figure 2).

To better assess the relationship between dietary phosphorus, magnesium, copper, selenium intake and kidney stones, we quadrupled-classified phosphorus, magnesium, copper, and selenium. After adjusting for all confounding variables, multivariate single-metal model logistic regression analysis revealed that dietary phosphorus, magnesium or copper intake was an independent risk factor for kidney stones. Compared with the lowest quartile, the ORs of kidney stones for each quartile of phosphorus intake were 0.977 (0.868–1.086, *p* = 0.605), 0.899 (0.801–1.010, *p* = 0.072) and 0.821 (0.727–0.929, *p* = 0.002), respectively; 0.874 (0.782–0.977, *p* = 0.018), 0.819 (0.730–0.919, *p* = 0.001), and 0.770 (0.683–0.868, *p* < 0.001) for each quartile of magnesium intake, respectively; 0.905 (0.808–1.011, *p* = 0.075), 0.880 (0.785–0.987, *p* = 0.028), and 0.853 (0.756–0.959, *p* = 0.009) for each quartile of copper intake, respectively (Table 2). However, in multivariate multi-metal logistic regression, only magnesium intake was associated with the risk of kidney stones (Supplementary Table S1).

**Table 2:** Multivariate single-metal model logistic regression the relationship between the heavy metals and the presence of kidney stone.

**Metals**	**Phosphorus**		**Magnesium**		**Copper**		**Selenium**	
	**aOR (95% CI)**	***P***	**aOR (95% CI)**	***P***	**aOR (95% CI)**	***P***	**aOR (95% CI)**	***P***
Overall		0.008		<0.001		0.041		0.133
Q1	Reference		Reference		Reference		Reference	
Q2	0.971 (0.868–1.086)	0.605	0.874 (0.782–0.977)	0.018	0.905 (0.808–1.011)	0.075	0.983 (0.879–1.100)	0.769
Q3	0.899 (0.801–1.010)	0.072	0.819 (0.730–0.919)	0.001	0.880 (0.785–0.987)	0.028	0.952 (0.849–1.068)	0.399
Q4	0.821 (0.727–0.929)	0.002	0.770 (0.683–0.868)	<0.001	0.853 (0.756–0.959)	0.009	0.872 (0.772–0.986)	0.029
Male		0.007		0.001		0.137		0.054
Q1	Reference		Reference		Reference		Reference	
Q2	1.026 (0.864–1.217)	0.773	0.901 (0.763–1.065)	0.222	0.937 (0.793–1.106)	0.441	0.972 (0.820–1.153)	0.747
Q3	0.991 (0.838–1.171)	0.911	0.925 (0.787–1.088)	0.347	0.919 (0.781–1.082)	0.310	0.951 (0.807–1.122)	0.554
Q4	0.810 (0.684–0.958)	0.014	0.736 (0.625–0.867)	<0.001	0.829 (0.704–0.976)	0.024	0.821 (0.696–0.968)	0.019
Female		0.063		0.001		0.343		0.670
Q1	Reference		Reference		Reference		Reference	
Q2	0.929 (0.799–1.080)	0.340	0.868 (0.746–1.010)	0.066	0.897 (0.768–1.048)	0.170	0.963 (0.828–1.120)	0.624
Q3	0.798 (0.675–0.943)	0.008	0.701 (0.591–0.831)	<0.001	0.875 (0.740–1.034)	0.116	0.917 (0.778–1.080)	0.299
Q4	0.958 (0.793–1.157)	0.653	0.932 (0.776–1.119)	0.449	0.968 (0.810–1.158)	0.726	1.024 (0.845–1.241)	0.809
BMI < 25.0 kg/m^2^		0.412		0.136		0.623		0.403
Q1	Reference		Reference		Reference		Reference	
Q2	0.919 (0.715–1.181)	0.509	0.897 (0.698–1.153)	0.396	0.924 (0.715–1.195)	0.546	1.112 (0.869–1.422)	0.399
Q3	0.949 (0.735–1.225)	0.688	0.842 (0.649–1.091)	0.193	0.934 (0.722–1.209)	0.604	0.922 (0.709–1.198)	0.544
Q4	0.794 (0.602–1.049)	0.105	0.728 (0.556–0.953)	0.021	0.837 (0.642–1.092)	0.190	0.913 (0.695–1.200)	0.515
BMI ≥ 25.0 kg/m^2^		0.019		0.001		0.173		0.199
Q1	Reference		Reference		Reference		Reference	
Q2	0.983 (0.866–1.114)	0.784	0.867 (0.766–0.982)	0.025	0.908 (0.801–1.030)	0.135	0.952 (0.839–1.080)	0.447
Q3	0.884 (0.776–1.007)	0.063	0.813 (0.715–0.924)	0.002	0.885 (0.778–1.007)	0.063	0.958 (0.843–1.089)	0.513
Q4	0.828 (0.722–0.949)	0.007	0.781 (0.683–0.893)	<0.001	0.874 (0.765–0.998)	0.047	0.864 (0.753–0.991)	0.036

CI, confidence interval; aOR, adjusted odds ratio.

Adjusted to: gender, age, race, marital status, education level, BMI, hypertension, diabetes mellitus, vigorous physical activities, moderate physical activities, blood urea nitrogen, creatinine, uric acid, and estimated glomerular filtration rate (eGFR).

Dietary copper enters the body and is absorbed by the digestive system, synthesized by the liver into copper cyanobrotein and released into the blood, which is mainly involved in the physiological functions of the body in the form of plasma copper cyanobrotein (20). Most of the endogenous copper is excreted into the gastrointestinal tract along with unabsorbed copper, and a small amount of copper is excreted through other routes (21). One study found that increased exposure of serum copper was significantly associated with the risk of non-alcoholic fatty liver disease (NAFLD) and was particularly prominent in women, middle age and participants with improved insulin resistance status, with a 97% increased risk of NAFLD in the highest quartile of copper compared to the lowest quartile (22). Gao et al. (23) found that serum copper levels in the highest quartile were associated with urge incontinence (aOR = 1.74, 95% CI 1.11–2.74), especially in women over 50 years of age, serum copper levels in the highest quartile were associated with urge incontinence (aOR = 1.74, 95% CI 1.57–5.49), any urinary incontinence (aOR = 1.97, 95% CI 1.19–3.27), mixed incontinence (aOR = 2.43, 95% CI 1.19–4.97), and moderate/severe incontinence (aOR = 2.37, 95% CI 1.06–5.31). In addition, Bagheri et al. (24) found that serum copper levels were higher in patients with atherosclerosis and positively correlated with the severity of atherosclerosis (*p* = 0.001). The above findings suggest that excess serum copper levels can also adversely affect the organism and that excessive copper intake should be limited for normal individuals as well.

**Figure 3:**
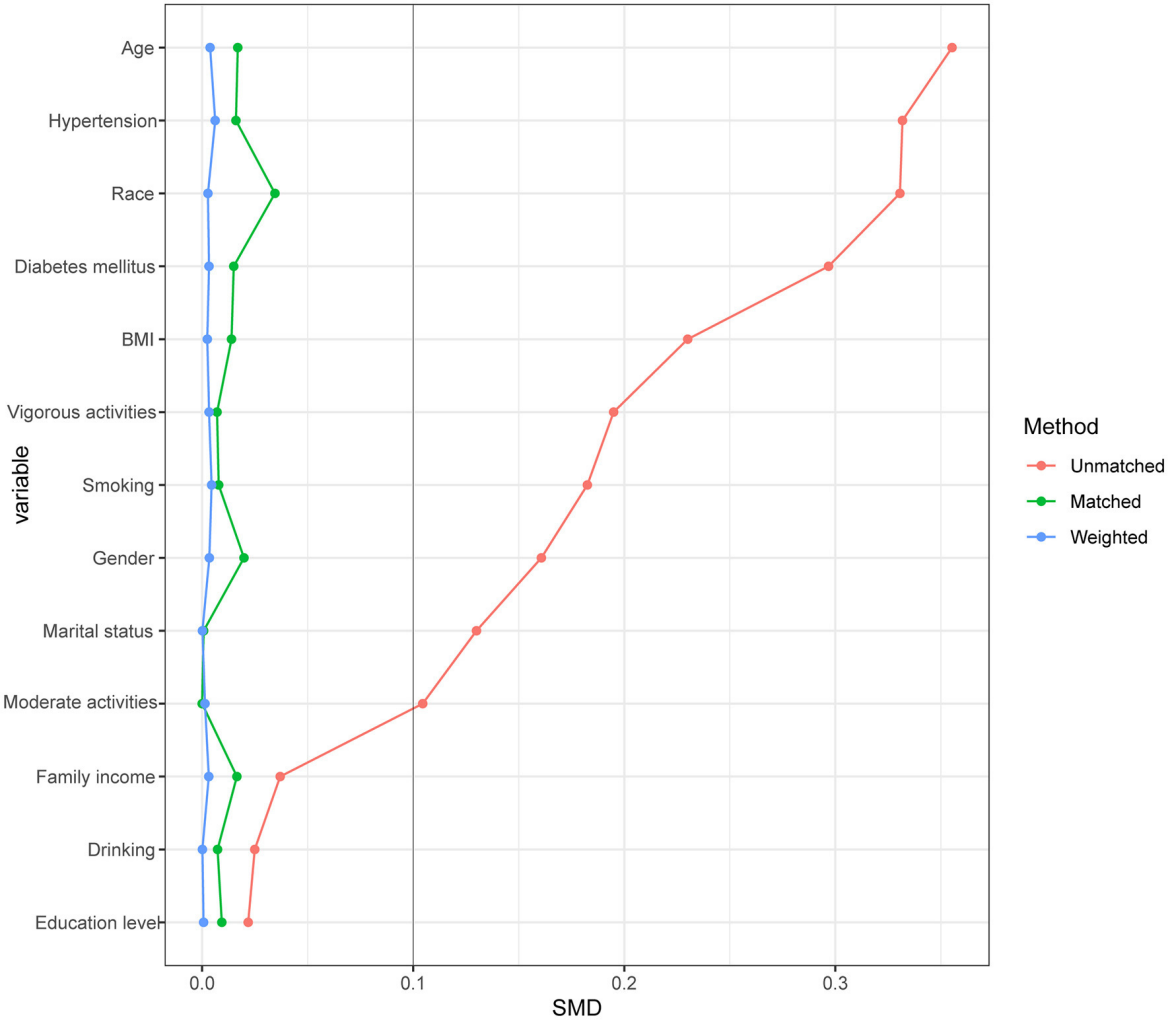
Propensity score matching analysis of the standardized mean difference results for the different variables.

**Figure 4:**
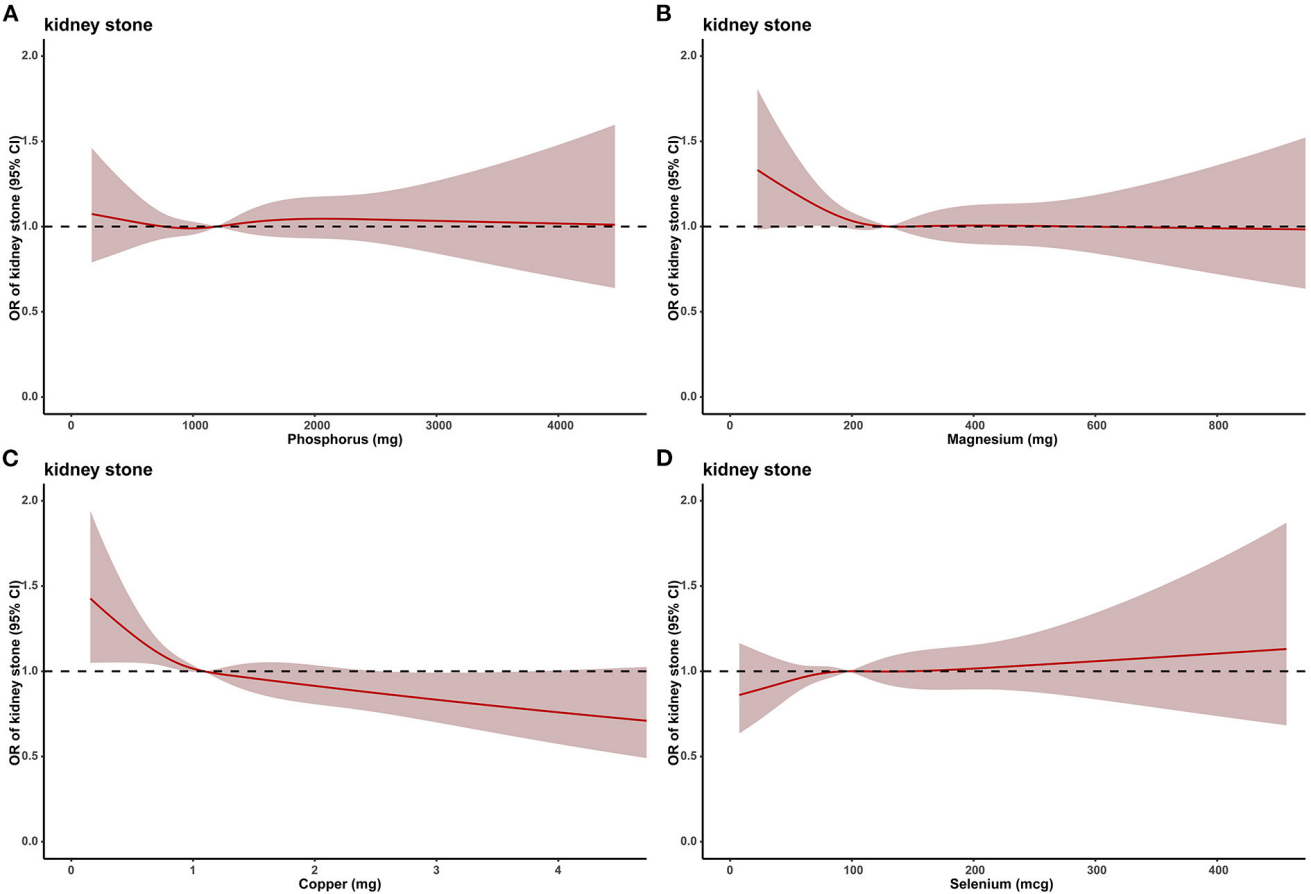
The dose–response analysis between dietary metal intake and presence of kidney stones after propensity score matching. **(A)** Phosphorus. **(B)** Magnesium. **(C)** Copper. **(D)** Selenium.

Due to the large difference in the number of stone formers and non-stone formers groups, we used a 1:1 PSM to correct for the difference between the two groups (Figure 3), 2,744 participants were included in both the stone formers and non-stone formers groups after the PSM. The clinicopathological characteristics of all participants after PSM are shown in Table 3. Subsequently, dose-response curves showed that only dietary copper (*p* = 0.001) intake was negatively and non-linearly associated with the risk of kidney stones (Figure 4). Multivariate single-metal model logistic regression results also demonstrated that dietary copper (*p* = 0.001) intake remained an independent risk factor for kidney stones, and the ORs for kidney stones in each quartile of copper intake compared to the lowest quartile were 0.877 (0.754–1.021, *p* = 0.092), 0.876 (0.751–1.022, *p* = 0.093) and 0.732 (0.626–0.856, *p* < 0.001) (Table 4). Moreover, multivariate multi-metal logistic regression showed that only copper intake was associated with the risk of kidney stones (Table 5).

**Table 3:** Baseline characteristics of NHANES participants between 2007 and 2018 after PSM.

**Characteristic**	**Total**	**None-stone formers**	**Stone formers**	***P*** **-value**
	**No. (%)**	**No. (%)**	**No. (%)**	
Total patients	5,488	2,744 (50.0)	2,744 (50.0)	
**Gender**				0.786
Male	3,056 (55.7)	1,523 (55.5)	1,533 (55.9)	
Female	2,432 (44.3)	1,221 (44.5)	1,211 (44.1)	
**Age**				0.459
<50 years	1,876 (34.2)	925 (33.7)	951 (34.7)	
≥50 years	3,612 (65.8)	1,819 (66.3)	1,793 (65.3)	
**Race**				0.783
Non-Hispanic white	3,059 (55.7)	1,542 (56.2)	1,517 (55.3)	
Non-Hispanic black	711 (13.0)	359 (13.1)	352 (12.8)	
Mexican American	697 (12.7)	343 (12.5)	354 (12.9)	
Other Hispanic	610 (11.1)	306 (11.2)	304 (11.1)	
Other	411 (7.5)	194 (7.1)	217 (7.9)	
**Education level**				0.991
Less than high school	1,363 (24.8)	683 (24.9)	680 (24.8)	
High school or equivalent	1,240 (22.6)	621 (22.6)	619 (22.6)	
College or above	2,885 (52.6)	1,440 (52.5)	1,445 (52.7)	
**Marital status**				0.155
Married	3,200 (58.3)	1,626 (59.3)	1,574 (57.4)	
Unmarried	2,288 (41.7)	1,118 (40.7)	1,170 (42.6)	
**Family income**				0.786
< $20,000	1,324 (24.1)	668 (24.3)	656 (23.9)	
≥$20,000	3,945 (71.9)	1,971 (71.8)	1,974 (71.9)	
Unknown	219 (4.0)	105 (3.8)	114 (4.2)	
**BMI (kg/m^2^)**				0.891
<25.0	1,062 (19.4)	533 (19.4)	529 (19.3)	
≥25.0	4,426 (80.6)	2,211 (80.6)	2,215 (80.7)	
**Hypertension**				0.666
Yes	2,768 (50.4)	1,376 (50.1)	1,392 (50.7)	
No	2,720 (49.6)	1,368 (49.9)	1,352 (49.3)	
**Diabetes mellitus**				0.096
Yes	1,219 (22.2)	603 (22.0)	616 (22.4)	
No	4,107 (74.8)	2,073 (75.5)	2,034 (74.1)	
Borderline	162 (3.0)	68 (2.5)	94 (3.4)	
**Smoking status**				0.084
Never	2,684 (48.9)	1,339 (48.8)	1,345 (49.0)	
Former	1,703 (31.0)	882 (32.1)	821 (29.9)	
Current	1,101 (20.1)	523 (19.1)	578 (21.1)	
**Drinking**				0.768
Yes	3,856 (70.3)	1,933 (70.4)	1,923 (70.1)	
No/Unknown	1,632 (29.7)	811 (29.6)	821 (29.9)	
**Vigorous recreational activities**				0.519
Yes	815 (14.9)	399 (14.5)	416 (15.2)	
No	4,673 (85.1)	2,345 (85.5)	2,328 (84.8)	
**Moderate recreational activities**				0.693
Yes	1,954 (35.6)	970 (35.3)	984 (35.9)	
No	3,534 (64.4)	1,774 (64.7)	1,760 (64.1)	
Blood urea nitrogen (mg/dL)	15.02, 6.96	14.74, 6.63	15.29, 7.27	0.003
Creatinine (mg/dL)	0.96, 0.60	0.95, 0.45	0.98, 0.72	0.057
Uric acid (mg/dL)	5.69, 1.50	5.73, 1.48	5.65, 1.52	0.042
eGFR [mL/(min·1.73 m^2^)]	75.36, 25.24	75.68, 24.85	75.05, 25.61	0.352
**Daily intake (M, SD)**				
Protein (mg)	79.46, 40.56	80.38, 41.18	78.53, 39.92	0.129
Moisture (g)	2.88, 1.48	2.88, 1.47	2.88, 1.50	0.959
Phosphorus (mg)	1,304.9, 642.0	1,301.9, 642.3	1,307.9, 641.9	0.727
Magnesium (mg)	285.38, 138.83	287.90, 138.12	282.86, 139.52	0.178
Iron (mg)	14.50, 8.26	14.58, 8.20	14.41, 8.33	0.461
Zinc (mg)	11.08, 6.68	11.30, 6.93	10.87, 6.43	0.018
Copper (mg)	1.23, 0.72	1.27, 0.75	1.20, 0.69	<0.001
Selenium (mcg)	109.18, 61.61	108.30, 60.76	110.06, 62.44	0.290

For categorical variables, *P*-values were analyzed by chi-square tests. For continuous variables, the *t*-test for slope was used in generalized linear models.

NHANES, National Health and Nutrition Examination Survey; BMI, body mass index; eGFR, estimated glomerular filtration rate; SD, standard deviation; PSM, propensity score matching.

**Table 4:** Multivariate single-metal model logistic regression the relationship between the heavy metals and the presence of kidney stone after PSM.

**Metals**	**Phosphorus**		**Magnesium**		**Copper**		**Selenium**	
	**aOR (95% CI)**	***P***	**aOR (95% CI)**	***P***	**aOR (95% CI)**	***P***	**aOR (95% CI)**	***P***
Overall		0.929		0.307		0.001		0.297
Q1	Reference		Reference		Reference		Reference	
Q2	1.051 (0.906–1.219)	0.512	0.903 (0.779–1.047)	0.177	0.877 (0.754–1.021)	0.092	1.134 (0.977–1.315)	0.098
Q3	1.023 (0.878–1.193)	0.770	0.903 (0.775–1.052)	0.191	0.876 (0.751–1.022)	0.093	1.113 (0.957–1.295)	0.165
Q4	1.016 (0.865–1.193)	0.849	0.865 (0.739–1.014)	0.073	0.732 (0.626–0.856)	<0.001	1.034 (0.881–1.213)	0.684
Male		0.680		0.129		<0.001		0.306
Q1	Reference		Reference		Reference		Reference	
Q2	1.032 (0.825–1.291)	0.783	0.884 (0.710–1.101)	0.270	0.792 (0.632–0.992)	0.042	1.135 (0.908–1.418)	0.265
Q3	1.024 (0.823–1.275)	0.830	0.930 (0.751–1.153)	0.510	0.822 (0.659–1.025)	0.082	1.127 (0.909–1.396)	0.276
Q4	0.926 (0.744–1.152)	0.491	0.784 (0.632–0.972)	0.026	0.618 (0.497–0.768)	<0.001	0.975 (0.787–1.207)	0.814
Female		0.265		0.261		0.827		0.562
Q1	Reference		Reference		Reference		Reference	
Q2	1.052 (0.860–1.287)	0.621	0.925 (0.756–1.133)	0.452	0.948 (0.769–1.169)	0.619	1.108 (0.906–1.356)	0.318
Q3	0.972 (0.778–1.214)	0.802	0.836 (0.667–1.047)	0.119	0.903 (0.723–1.128)	0.371	1.075 (0.864–1.338)	0.517
Q4	1.268 (0.976–1.647)	0.075	1.074 (0.838–1.378)	0.572	0.927 (0.731–1.176)	0.535	1.192 (0.917–1.550)	0.190
BMI <25.0 kg/m^2^		0.888		0.330		0.291		0.480
Q1	Reference		Reference		Reference		Reference	
Q2	0.903 (0.642–1.269)	0.556	1.097 (0.776–1.550)	0.601	1.050 (0.737–1.496)	0.788	1.244 (0.885–1.747)	0.209
Q3	0.892 (0.627–1.269)	0.524	0.935 (0.654–1.336)	0.711	1.024 (0.717–1.461)	0.897	1.089 (0.762–1.554)	0.641
Q4	0.871 (0.592–1.280)	0.481	0.784 (0.546–1.127)	0.190	0.771 (0.538–1.104)	0.155	0.961 (0.664–1.392)	0.834
BMI ≥ 25.0 kg/m^2^		0.784		0.356		0.003		0.474
Q1	Reference		Reference		Reference		Reference	
Q2	1.091 (0.924–1.287)	0.305	0.870 (0.738–1.025)	0.097	0.843 (0.712–0.998)	0.048	1.113 (0.943–1.313)	0.207
Q3	1.057 (0.891–1.254)	0.526	0.898 (0.758–1.065)	0.217	0.846 (0.712–1.005)	0.057	1.125 (0.951–1.330)	0.169
Q4	1.051 (0.880–1.257)	0.581	0.884 (0.741–1.055)	0.172	0.716 (0.601–0.853)	<0.001	1.050 (0.879–1.255)	0.593

CI, confidence interval; aOR, adjusted odds ratio; PSM, propensity score matching.

Adjusted to: gender, age, race, marital status, education level, BMI, hypertension, diabetes mellitus, vigorous physical activities, moderate physical activities, blood urea nitrogen, creatinine, uric acid, and estimated glomerular filtration rate (eGFR).

**Table 5:** Multivariate multi-metal model logistic regression of the relationship between heavy metals and kidney stone after PSM.

**Metals**	**Phosphorus**		**Magnesium**		**Copper**		**Selenium**	
	**aOR (95% CI)**	***P***	**aOR (95% CI)**	***P***	**aOR (95% CI)**	***P***	**aOR (95% CI)**	***P***
Overall		0.504		0.599		<0.001		0.246
Q1	Reference		Reference		Reference		Reference	
Q2	1.109 (0.931–1.321)	0.247	0.923 (0.768–1.110)	0.394	0.827 (0.693–0.986)	0.034	1.163 (0.990–1.366)	0.067
Q3	1.150 (0.924–1.430)	0.210	0.989 (0.786–1.245)	0.927	0.760 (0.616–0.938)	0.011	1.185 (0.983–1.429)	0.076
Q4	1.224 (0.936–1.600)	0.139	1.059 (0.800–1.402)	0.689	0.581 (0.454–0.744)	<0.001	1.149 (0.922–1.433)	0.216

CI, confidence interval; aOR, adjusted odds ratio; PSM, propensity score matching.

Adjusted to: gender, age, race, marital status, education level, BMI, hypertension, diabetes mellitus, vigorous physical activities, moderate physical activities, blood urea nitrogen, creatinine, uric acid, estimated glomerular filtration rate (eGFR), phosphorus, magnesium, copper and selenium.

## Discussion

As a common kidney disease, the high incidence and recurrence rate of kidney stones bring serious impact on patients' life, and cause huge economic burden. Therefore, it is of great clinical and social significance to explore the factors associated with kidney stones and to identify risk factors and protective factors for kidney stones in advance. In this study, we first investigated the dietary trace metals associated with kidney stones, and found that increased dietary intake of phosphorus, magnesium, and copper were associated with lower odds of kidney stones, and this finding was confirmed by the dose-response curves. Subsequently, multivariate logistic regression also revealed that dietary phosphorus, magnesium, and copper were independent protective factors for kidney stones. To exclude as much as possible the influence of other confounding variables and to test the correctness of the results, we used PSM analysis to adjust for differences between the stone formers and non-stone formers groups. Subsequent dose-response curves as well as multivariate logistic regression confirmed that dietary copper intake may be a protective factor for kidney stones.

Copper, an essential trace element, is widely distributed in tissues and organs such as the liver, kidney and brain, and the body meets its requirement for copper mainly through daily dietary intake (25). Copper is involved in energy metabolism, immune function, antioxidant and energy metabolism in the body (26). Numerous studies have shown that deficiencies in dietary copper intake are associated with cardiovascular disease, central nervous system diseases, digestive system diseases, and skeletal system diseases (13, 14, 27). Li et al. (28) included 14,834 NHANES participants from 2009 to 2014 and found that dietary intake of zinc, iron, copper, and selenium were negatively associated with depression, and similar study has found that dietary intake of zinc, copper, and selenium was associated with cognitive performance (29). Yang et al. (30) found a non-linear negative association between dietary copper intake and stroke by analyzing clinical information from 10,550 participants in the NHANES database from 2013 to 2018. The above results suggest that increased dietary copper intake appears to be a protective factor against disease progression.

Recently, Tsvetkov et al. (17) found that intracellular copper accumulation can cause direct binding of copper to lipid acylated components of the tricarboxylic acid (TCA) cycle, which subsequently leads to aggregation of lipid acylated proteins and loss of Fe-S cluster proteins, ultimately leading to cell death. Tsvetkov.'s study has led researchers to pay increasing attention to the role of copper in living organisms. To date, there has been only one study of copper intake and risk of kidney stones in adults. Ferraro et al. (12) studied participants in the Health Professionals Follow-up Study (HPFS) and the Nurses' Health Study (NHS) I and II and found no association between dietary copper intake and kidney stone risk, but total copper intake was associated with a higher risk of kidney stones in the NHS I cohort (OR = 1.14, 95% CI 1.02–1.28). However, in our study, we found that dietary copper intake was associated with a lower risk of kidney stones, and the same results were also obtained after 1:1 PSM analysis. We speculate that the conflicting results may be due to the following reasons: first, copper has two aspects; it is both an antioxidant and a pro-oxidant. One study found that either excessive or insufficient copper intake increased susceptibility to atherosclerosis in rabbits, reflecting the bidirectional relationship between copper and atherosclerosis (31). Second, the year of sample collection, sample size, and type of variables adjusted for differed for each study. Therefore, further large samples are needed to elucidate the relationship between dietary copper intake and the risk of kidney stones.

There are several strengths deserve to be noted in our study. First, the sample of this study was large enough that the participants were representative of the national population of the United States, and the findings warrant generalization. Second, we used multivariate logistic regression analysis, to adjust for other confounding variables in our statistical analysis. Finally, to verify the correctness of the results, we also conducted a 1:1 PSM analysis, which was used to offset possible effects caused by gaps in the sample data. However, our analysis has some potential limitations. First, our study is a cross-sectional retrospective analysis with its inherent limitations. Second, although adjustments were made for some confounding factors, the effects of other unknown factors could not be excluded. Finally, the type of kidney stones was not available, and different stone types may have different results.

## Conclusion

In conclusion, this study suggests that dietary copper intake was significantly and negatively associated with kidney stones in NHANES participants and that dietary copper intake may play an important role in the prevention of kidney stones in future clinical practice.

## Data availability statement

The raw data supporting the conclusions of this article will be made available by the authors, without undue reservation.

## Ethics statement

This study was reviewed and approved by the NHANES Committee. Written informed consent was obtained from all the patients/participants for their participation in this study and for the publication of any potentially identifiable images or data included in this article, following the Declaration of Helsinki.

## Author contributions

SC, WM, YC, and MC designed the research. WZ, CW, JW, and WM performed the research and analyzed the results. WZ and CW wrote the paper. JW, SC, WM, YC, and MC edited the manuscript and provided the critical comments. All authors read and approved the final manuscript.

## Funding

This work was supported by National Natural Science Foundation of China (82070773), Natural Science Foundation of Jiangsu Provincial (BK20201271), Jiangsu Provincial Key Research and Development Program (BE2019751), Innovative Team of Jiangsu Provincial (2017ZXKJQW07), and the National Key Research and Development Program of China (SQ2017YFSF090096).

## Conflict of interest

The authors declare that the research was conducted in the absence of any commercial or financial relationships that could be construed as a potential conflict of interest.

## Publisher's note

All claims expressed in this article are solely those of the authors and do not necessarily represent those of their affiliated organizations, or those of the publisher, the editors and the reviewers. Any product that may be evaluated in this article, or claim that may be made by its manufacturer, is not guaranteed or endorsed by the publisher.

## Supplementary material

Supplementary Figure S1 Spearman's rank correlation coefficients of six trace elements.

Supplementary Table S1 Multivariate multi-metal model logistic regression the relationship between the heavy metals and the presence of kidney stone.
