# Supplementary figures and images for: Dietary copper intake and the prevalence of kidney stones among adult in the United States: A propensity score matching study
Source: Front Public Health. 2022 Aug 30;10:973887. doi: 10.3389/fpubh.2022.973887 (PMC9469499; doi:10.3389/fpubh.2022.973887)

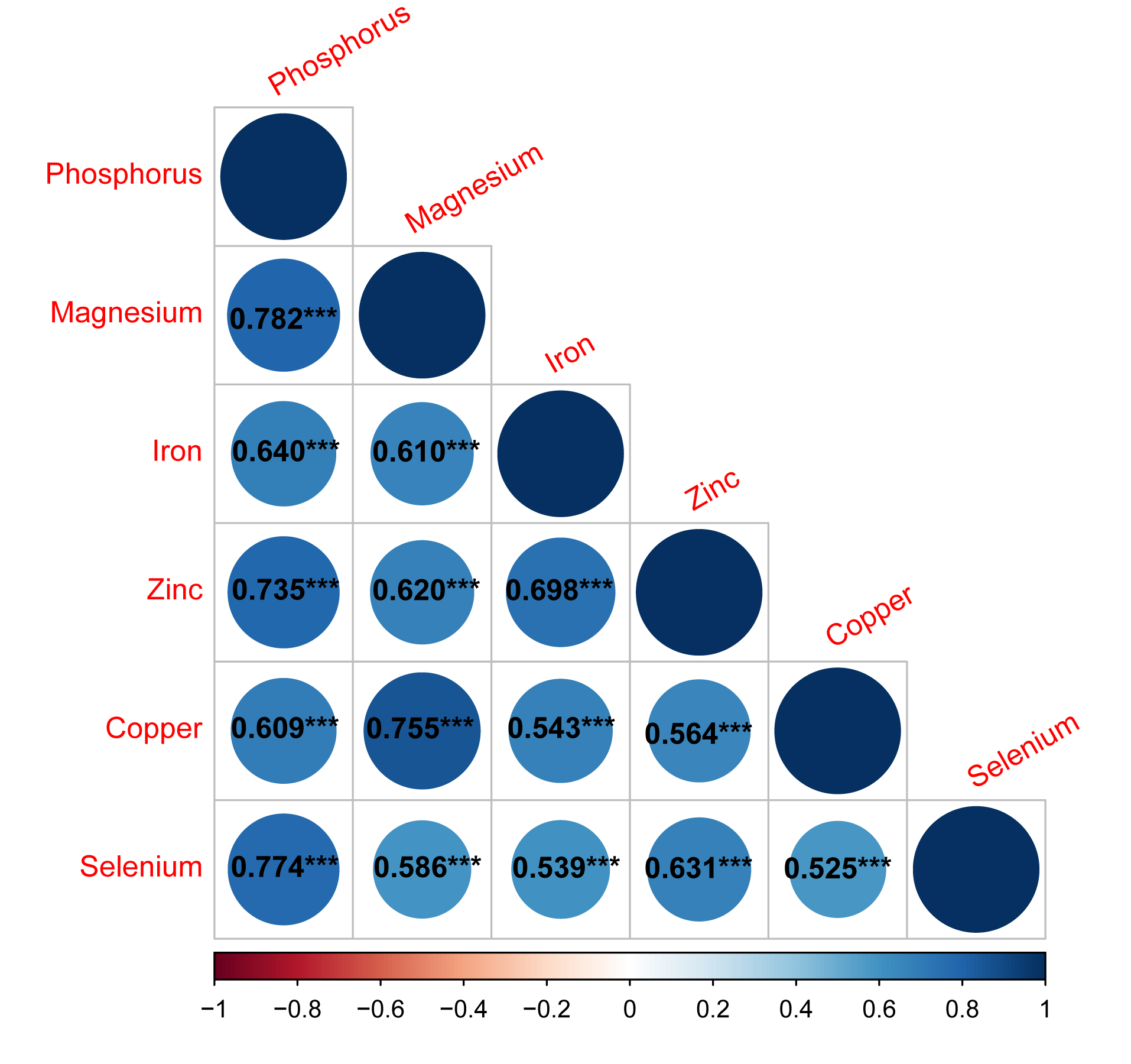

Supplement: Supplementary Figure S1 — Spearman's rank correlation coefficients of six trace elements. [file Image_1.JPEG]
