# Supplementary material for: Dietary copper intake and the prevalence of kidney stones among adult in the United States: A propensity score matching study
Source: Front Public Health. 2022 Aug 30;10:973887. doi: 10.3389/fpubh.2022.973887 (PMC9469499; doi:10.3389/fpubh.2022.973887)
Supplement: Supplementary Table S1 — Multivariate multi-metal model logistic regression the relationship between the heavy metals and the presence of kidney stone. [file Table_1.DOCX]

**Table S1**. Multivariate multi-metal model logistic regression the relationship between the heavy metals and the presence of kidney stone.

| **Metals** | **Phosphorus** | | **Magnesium** | | **Copper** | | **Selenium** | |
| --- | --- | --- | --- | --- | --- | --- | --- | --- |
|  | **aOR (95% CI)** | **P** | **aOR (95% CI)** | **P** | **aOR (95% CI)** | **P** | **aOR (95% CI)** | **P** |
| Overall |  | 0.772 |  | 0.007 |  | 0.330 |  | 0.929 |
| Q1 | Reference |  | Reference |  | Reference |  | Reference |  |
| Q2 | 1.037 (0.908-1.185) | 0.589 | 0.836 (0.728-0.962) | 0.012 | 1.018 (0.890-1.163) | 0.797 | 1.023 (0.905-1.156) | 0.717 |
| Q3 | 1.010 (0.858-1.189) | 0.905 | 0.758 (0.637-0.902) | 0.002 | 1.100 (0.938-1.290) | 0.239 | 1.034 (0.899-1.190) | 0.640 |
| Q4 | 0.960 (0.785-1.173) | 0.688 | 0.699 (0.566-0.863) | 0.001 | 1.169 (0.969-1.410) | 0.104 | 0.998 (0.846-1.178) | 0.985 |

Abbreviations: CI: confidence interval. aOR: adjusted odds ratio.

adjusted to: gender, age, race, marital status, education level, BMI, hypertension, diabetes mellitus, vigorous physical activities, moderate physical activities, blood urea nitrogen, creatinine, uric acid, estimated glomerular filtration rate (eGFR), phosphorus, magnesium, copper and selenium.
